# Density Functional Theory and Information-Theoretic Diagnostics of Quantum Phase Transitions

**DOI:** 10.3390/e28020170

**Published:** 2026-02-01

**Authors:** Elvira Romera, Ágnes Nagy

**Affiliations:** 1 Departamento de Física Atómica, Molecular y Nuclear and Instituto Carlos I de Física Teórica y Computacional, Universidad de Granada, Fuentenueva s/n, 18071 Granada, Spain; eromera@ugr.es; 2 Department of Theoretical Physics, University of Debrecen, H-4002 Debrecen, Hungary

**Keywords:** density functional theory, quantum phase transitions, Rényi entropy, Fisher information, relative Rényi entropy, fidelity susceptibility

## Abstract

Within density functional theory (DFT), where the density is the fundamental variable, quantum phase transitions (QPTs) can be formulated through a Hamiltonian $\hat{H}={\hat{H}}_{0}+\sum_{i}\xi_{i}{\hat{A}}_{i}$, such that the control parameters $\left\{\xi_{i}\right\}$ are in bijective correspondence (in the nondegenerate case) with the “densities” $a_{i}=\langle {\hat{A}}_{i}\rangle$, and the functional $Q(\left\{a_{i}\right\})$ acts as the Legendre transform of the energy; this structure even permits the use of Rényi entropy (for a given order) as an alternative control parameter, while degeneracy can be handled via a subspace density. On this foundation, information-theoretic measures provide sensitive diagnostics of criticality: fidelity and its susceptibility χ, Fisher information, relative Rényi entropy, and the Kullback–Leibler divergence are locally linked by $R^{q}\;\approx q\;I_{KL}\;\approx 2q\;\chi \;{\left(\delta \lambda \right)}^{2}$, revealing their proportionality in the small-parameter-shift regime. Applied to the Dicke model, numerical analyses show that fidelity exhibits pronounced curvature or divergence near $\lambda_{c}=\sqrt{\omega \omega_{0}}/2$ and that the response sharpens with increasing *j*, corroborating that these information measures capture QPTs with precision within the DFT framework.

## 1. Introduction

The interest in information-theoretic measures has grown steadily over the last decade. Among them, the entropies of *Shannon* [1], *Fisher* [2], and *Rényi* [3] have proved particularly versatile and effective across a wide spectrum of physical contexts: from the analysis of *entanglement* [4] and *quantum communication protocols* [5] to studies of *quantum correlations*, *revivals*, and *localization* phenomena [6]. They have also been successfully applied to the investigation of *atomic and molecular* properties [7,8,9,10,11] and to the scrutiny of *quantum phase transitions* (QPTs) [12,13].

Moreover, several works have clearly demonstrated that quantum information measures—Rényi and Wehrl entropies, Fisher information, participation ratios, complexity measures, and fidelity-based quantities—provide a unified and highly sensitive characterization of both quantum and topological phase transitions [12,14,15]. On the one hand, these tools allow one to identify and classify quantum phase transitions in paradigmatic many-body models, such as the Dicke model, the vibron model, and the Lipkin–Meshkov–Glick model, by revealing sharp changes in the localization properties and internal structure of the wave functions in phase space [12,13,16,17,18]. On the other hand, the same information-theoretic characterizations have been successfully extended to gapped two-dimensional Dirac materials, where these quantities act as sharp markers of transitions between topological and trivial insulating phases, as well as of changes in the associated topological invariants.

In classical phase transitions the physical properties of a system change suddenly owing to the change in a parameter (e.g., the temperature). These phenomena are induced by classical fluctuations (e.g., thermal fluctuations). Quantum phase transitions (QPTs), on the other hand, appear at the absolute zero temperature. The dramatic change in the properties of the ground-state system has taken place due to the so-called quantum fluctuations induced by quantum uncertainty. That is, from a physical standpoint, a QPT constitutes the natural extension of the classical notion of a phase transition to the *zero-temperature* limit: it is *quantum fluctuations*, tuned by control parameters, that drive abrupt changes in the properties of the system [19]. Within this setting, *density functional theory* (DFT) [20] provides a particularly suitable framework [14,21,22].

An appropriate form of DFT as a means to study QPTs was proposed by Wu et al. [21], introducing an analogue of the standard DFT density: a *new density* that determines the control parameters, in correspondence with the DFT *external potential*.

In the work we published more than a decade ago, in the *nondegenerate* case we established a rigorous bijection between the density and the control parameters. Moreover, any strictly monotonic function of the external potential induces a new density which, in turn, fixes a new control parameter. A particularly relevant consequence is the existence of a one-to-one mapping between the *Rényi entropy* of a given order and the control parameter, thereby enabling the use of the former as an *alternative control parameter* [23]. *Degenerate* cases can be handled in an analogous manner by means of the *subspace density* [22].

Building on this foundation, we examined systematically the connection between the *relative Rényi entropy* [23] and the *fidelity susceptibility* [15]. It was shown that, to an excellent approximation, the former is *proportional* to the latter and that the relative Rényi entropy coincides with the *Kullback–Leibler entropy* [24] multiplied by the Rényi parameter. These identities endow information-theoretic measures with operational content for diagnosing QPTs. Finally, it is worth noting that the Kullback–Leibler entropy has been used recently to *quantify chemical reactivity* [25,26], further underscoring the cross-disciplinary scope of these tools in theoretical physics and chemistry. In this article we provide an overview of our contributions that intertwine density functional theory with quantum phase transitions and information theory.

## 2. Density Functional Theory, Rényi Entropy and Quantum Phase Transition

DFT has been extended to treat quantum phase transition [14,21,22]. To enlighten the essence of the generalization it is worth recollecting the fundamental theorems of traditional DFT. The original form of the Hamiltonian is

(1) $$\begin{array}{c} \hat{H}=\hat{T}+{\hat{V}}_{ee}+\sum_{i=1}^{N}v\left(r_{i}\right)\;, \end{array}$$

where $\hat{T}$ and ${\hat{V}}_{ee}$ are the kinetic energy and the electron–electron energy operators. According to the Hohenberg–Kohn theorems [20] there is a one-to-one map between the density ϱ and the external potental v(r), and there is a variational principle for the energy functional

(2) E=F+∫ϱ(r)v(r)dr.

The universal functional F[ϱ] can be defined with the constraint search [27]

(3) $$\begin{array}{c} F\left[\varrho \right]=\min_{\Psi \to \varrho }\langle \Psi |\hat{T}+{\hat{V}}_{ee}|\Psi \rangle \;. \end{array}$$

That is, the ground-state energy is sought in two steps:

(4) $$\begin{array}{ccc} E_{0} & = & \underset{\Psi }{\mathrm{Min}}\langle \Psi |\hat{T}+{\hat{V}}_{ee}+\sum_{i=1}^{N}v\left(r_{i}\right)|\Psi \rangle \\ & = & \underset{\varrho }{\mathrm{Min}}\left\{\underset{\Psi \to \varrho }{\mathrm{Min}}\langle \Psi |\hat{T}+{\hat{V}}_{ee}+\sum_{i=1}^{N}v\left(r_{i}\right)|\Psi \rangle \right\}\\ & = & \underset{\varrho }{\mathrm{Min}}\left\{\underset{\Psi \to \varrho }{\mathrm{Min}}\left[\langle \Psi |\hat{T}+{\hat{V}}_{ee}|\Psi \rangle +\int v\left(r\right)\varrho \left(r\right)dr\right]\right\}\;. \end{array}$$

The inner minimization for wave functions yielding the given ϱ produce F[ϱ]. The functional derivative of *F* leads to the Euler equation up to a constant

(5) $$\begin{array}{c} \frac{\delta F[\varrho ]}{\delta \varrho }=-v\left(r\right). \end{array}$$

In degenerate case it is favorable using density matrix and subspace density. Consider the subspace *S* spanned by the eigen functions $\Psi_{\gamma }$ of the Hamiltonian $\hat{H}$

(6) $$\begin{array}{c} \hat{H}|\Psi_{\gamma }\rangle =E|\Psi_{\gamma }\rangle \;\;\left(\gamma =1,2,\ldots ,g\right)\;, \end{array}$$

where *g* is the degeneracy. The density matrices in the subspace *S* is defined as

(7) $$\begin{array}{c} \hat{D}=N\sum_{\gamma =1}^{g}w_{\gamma }|\Psi_{\gamma }\rangle \langle \Psi_{\gamma }|\;, \end{array}$$

where the weighting factors $w_{\gamma }$ satisfy the conditions

(8) $$\begin{array}{c} 1=\sum_{\gamma =1}^{g}w_{\gamma } \end{array}$$

and

(9) $$\begin{array}{c} w_{\gamma }\geq 0\;. \end{array}$$

The subspace density is defined as

(10) $$\begin{array}{c} \varrho =N\sum_{\gamma =1}^{g}w_{\gamma }\int {\left|\Psi_{\gamma }\right|}^{2}ds_{1}dx_{2}\ldots dx_{N}\;, \end{array}$$

where the integration is over the spatial coordinates of N−1 particles, and there is a sum over all spin coordinates. Here the usual notation is used where x stands for both the coordinates and the spin.

The energy is the functional of the subspace density, and the constrained search can be applied as

(11) $$\begin{array}{ccc} E[\varrho ] & = & \min_{S}\sum_{\gamma =1}^{g}w_{\gamma }\langle \Psi_{\gamma }|\hat{H}|\Psi_{\gamma }\rangle \\ & = & \min_{\varrho }\left\{\min_{S\to \varrho }\sum_{\gamma =1}^{g}w_{\gamma }\langle \Psi_{\gamma }|\hat{H}|\Psi_{\gamma }\rangle \right\}\\ & = & \min_{\varrho }\left\{F[\varrho ]+\int \varrho (r)v(r)dr\right\}\;, \end{array}$$

where F[ϱ] is given by

(12) $$\begin{array}{c} F\left[\varrho \right]=\min_{S\to \varrho }tr\left\{\hat{D}\left(\hat{T}+{\hat{V}}_{ee}\right)\right\}\;. \end{array}$$

The variational principle states that

(13) $$\begin{array}{ccc} & & F\left[\varrho \right]+\int \varrho \left(r\right)v\left(r\right)dr=\min_{S\to \varrho }tr\left\{\hat{D}\left(\hat{T}+{\hat{V}}_{ee}\right)\right\}+\\ & & \int \varrho \left(r\right)v\left(r\right)dr=\min_{S\to \varrho }tr\left\{\hat{D}\left(\hat{T}+\hat{V}+{\hat{V}}_{ee}\right)\right\}\geq E_{0}. \end{array}$$

So, the second Hohenberg–Kohn theorem has the form

(14) $$\begin{array}{c} F\left[\varrho \right]+\int \varrho \left(r\right)v\left(r\right)dr\geq E_{0}. \end{array}$$

There is an equality iff the trial density ϱ is equal to the true subspace density. The functional derivative of *F* gives the external potential up to a constant

(15) $$\begin{array}{c} \frac{\delta F[\varrho ]}{\delta \varrho }=-v\left(r\right). \end{array}$$

Turning to the extension of DFT to quantum phase transitions [21] it is more convenient taking the Hamiltonian as

(16) $$\hat{H}={\hat{H}}_{0}+\sum_{i}\xi_{i}{\hat{A}}_{i}$$

instead of the form (1). ${\hat{H}}_{0}$ and ${\hat{A}}_{i}$ are known Hermitian operators. $\xi_{i}$ are the control parameters associated with ${\hat{A}}_{i}$ (Observe that taking the index *i* continuous the original DFT is recovered). Consider first the non-degenerate case. Denote the expectation value of ${\hat{A}}_{i}$ as

(17) $$a_{i}=\langle \Psi |{\hat{A}}_{i}|\Psi \rangle$$

and apply the constraint search (similarly as in Equation (3)) to define the functional

(18) $$\begin{array}{c} Q\left(\left\{a_{i}\right\}\right)=\min_{\Psi \to \{a_{i}\}}\langle \Psi |{\hat{H}}_{0}|\Psi \rangle \;. \end{array}$$

That is, $\left\{a_{i}\right\}$ corresponds to ϱ, and $\left\{\xi_{i}\right\}$ corresponds to *v*. After completing the constraint search

(19) $$\begin{array}{ccc} E & = & \underset{\Psi }{\mathrm{Min}}\langle \Psi |\hat{H}|\Psi \rangle \\ & = & \underset{\left\{a_{i}\right\}}{\mathrm{Min}}\left\{\underset{\Psi \to \{a_{i}\}}{\mathrm{Min}}\langle \Psi |{\hat{H}}_{0}|\Psi \rangle +\sum_{i}\xi_{i}a_{i}\right\}, \end{array}$$

the total energy is

(20) $$E\left(\xi_{1},\ldots ,\xi_{M}\right)=Q\left(a_{1},\ldots ,a_{M}\right)+\sum_{i}\xi_{i}a_{i}.$$

Therefore, *Q* is the Legendre transform of *E*

(21) $$Q\left(a_{1},\ldots ,a_{M}\right)=E-\sum_{i}\xi_{i}a_{i}.$$

$a_{i}$ can be obtained from the Hellmann–Feynman theorem [21]

(22) $$\frac{\partial E}{\partial \xi_{i}}=\langle \Psi |\frac{\partial \hat{H}}{\partial \xi_{i}}|\Psi \rangle =\langle \Psi |{\hat{A}}_{i}|\Psi \rangle =a_{i}.$$

We proved [14] that there is a one-to-one map between the non-degenerate wave function Ψ and the $\left\{\xi_{i}\right\}$. The first Hohenberg–Kohn theorem stands [14,21], namely, for a nondegenerate ground state there is a one-to-one map between $\left\{a_{i}\right\}$ and $\left\{\xi_{i}\right\}$, that is, the new *density* and the new *external potential*. The second Hohenberg–Kohn theorem also holds:

(23) $$Q\left(a_{1},\ldots ,a_{M}\right)+\sum_{i}\xi_{i}a_{i}\geq E_{gs},$$

where $E_{gs}$ is the ground-state energy. Equality iff $\left\{a_{i}\right\}$ is the ground-state *density*.

Observe that the Hamiltonian (16) can be recasted as

(24) $$\hat{H}={\hat{H}}_{0}+\sum_{i}\xi_{i}{\hat{A}}_{i}={\hat{H}}_{0}+\sum_{i}\zeta_{i}{\hat{B}}_{i}.$$

That is, another set of control parameters are taken and the corresponding *density* is

(25) $$b_{i}=\langle \Psi |{\hat{B}}_{i}|\Psi \rangle .$$

Obviously, ${\hat{A}}_{i}$ and ${\hat{B}}_{i}$ and their expectation values are related: ${\hat{B}}_{i}=\xi_{i}/\zeta_{i}{\hat{A}}_{i}$ and between $a_{i}$ and $b_{i}$: $b_{i}=\xi_{i}/\zeta_{i}a_{i}$ (Provided that $\zeta_{i}\neq 0$).

It has been proven [14] that, if $\zeta_{i}=f_{i}\left(\xi_{i}\right)$ and $f_{i}$ are strictly monotonous functions, there is a one-to-one map between the non-degenerate wave function Ψ and the *external potential* $\left\{\zeta_{i}\right\}$. Consequently, if $\zeta_{i}=f_{i}\left(\xi_{i}\right)$ and $f_{i}$ are strictly monotonous functions, there is a one-to-one map between the *density* $b_{i}$ and the *external potential* $\left\{\zeta_{i}\right\}$.

The variational principle can also be expressed with the set of control parameters $\zeta_{i}$:

(26) $$Q\left(b_{1},\ldots ,b_{M}\right)+\sum_{i}\zeta_{i}b_{i}\geq E_{gs}.$$

Equality iff $\left\{b_{i}\right\}$ is the new ground-state *density*.

In the degenerate case the expectation value of ${\hat{A}}_{i}$ is given by

(27) $$a_{i}=tr\left\{\hat{D}{\hat{A}}_{i}\right\}.$$

The constrained search provides

(28) $$\begin{array}{c} Q\left(\left\{a_{i}\right\}\right)=\underset{S\to \{a_{i}\}}{\mathrm{Min}}tr\left\{\hat{D}{\hat{H}}_{0}\right\}=\underset{\hat{D}\to \left\{a_{i}\right\}}{\mathrm{Min}}tr\left\{\hat{D}{\hat{H}}_{0}\right\}\;. \end{array}$$

Observe that $\left\{a_{i}\right\}$ correspond to the *subspace density* in the original DFT, while $\left\{\xi_{i}\right\}$ corresponds to the *external potential*. The steps of the constrained search give

(29) $$\begin{array}{ccc} E & = & \underset{\left\{a_{i}\right\}}{\mathrm{Min}}\left[\underset{S\to \{a_{i}\}}{\mathrm{Min}}tr\left\{\hat{D}\hat{H}\right\}\right]\\ & = & \underset{\left\{a_{i}\right\}}{\mathrm{Min}}\left[\underset{S\to \{a_{i}\}}{\mathrm{Min}}tr\left\{\hat{D}{\hat{H}}_{0}\right\}]+\sum_{i}\xi_{i}a_{i}\right\}\\ & = & \underset{\left\{a_{i}\right\}}{\mathrm{Min}}\left[Q\left(a_{1},\ldots ,a_{M}\right)+\sum_{i}\xi_{i}a_{i}\right]. \end{array}$$

Observe that *Q* is the Legendre transform of *E* as in the non-degenerate case (Equation (21)).

We proved the following theorem: If the inverse of the operator $\hat{P}=\sum_{i}c_{i}{\hat{A}}_{i}-c_{0}$ exits for any real $c_{0}$ and $c_{i}$, there is a one-to-one map between the subspace *S* spanned by the degenerate wave functions $\Psi_{\gamma }\left(\gamma =1,2,\ldots ,g\right)$ and the *external potential* $\left\{\xi_{i}\right\}$.

Recasting again the Hamiltonian as in Equation (16), where ${\hat{B}}_{i}={\hat{A}}_{i}\xi_{i}/\zeta_{i}$, $b_{i}=tr\left\{\hat{D}{\hat{B}}_{i}\right\}=a_{i}\xi_{i}/\zeta_{i}$ and $\zeta_{i}\neq 0$, we proved the theorem: If $\zeta_{i}=f_{i}\left(\xi_{i}\right)$ and $f_{i}$ are strictly monotonous functions, there is a one-to-one map between the subspace *S* and the *external potential* $\left\{\zeta_{i}\right\}$. $\zeta_{i}$ are the new control parameters associated with the new *density* $b_{i}$. The first Hohenberg–Kohn theorem holds in the degenerate case too: There is a one-to-one map between the *density* $a_{i}$ and the *external potential* $\left\{\xi_{i}\right\}$.

The second Hohenberg–Kohn theorem stands as

(30) $$Q\left(a_{1},\ldots ,a_{M}\right)+\sum_{i}\xi_{i}a_{i}\geq E_{gs},$$

where $E_{gs}$ is the ground-state energy of the Hamiltonian (16). Equality holds iff $\left\{a_{i}\right\}$ is the ground-state *density*. Or,

(31) $$Q\left(b_{1},\ldots ,b_{M}\right)+\sum_{i}\zeta_{i}b_{i}\geq E_{gs}.$$

Equality holds iff $\left\{b_{i}\right\}$ is the new ground-state *density*.

Wu et al. [21] established a link between entanglement and QPT via DFT. We studied the relationship of the Rényi entropy and the control parameter.

Let *f* be a (normalized) probability density or mass function on an appropriate measure space. The Rényi entropy of order α>0, α≠1, is defined by

(32) $$R_{f}^{\alpha }=\frac{1}{1-\alpha }\;\ln\;\int f^{\alpha }\left(r\right)\;dr.$$

This one-parameter family interpolates different notions of uncertainty: for α→0 it emphasizes support size; for α=2 it relates to the inverse participation ratio; and as α→∞ it focuses on the maximum of *f* (min-entropy). In the limit α→1, one recovers the Shannon entropy

(33) $$S_{f}=-\;\int f\left(r\right)\;\ln f\left(r\right)\;dr.$$

The parameter α thus provides tunability to weight tails or peaks of the distribution, which is useful near critical phenomena where probability mass may concentrate or delocalize.

While Wu et al. [21] used the new *density* in the expressions of the entanglement measures, we found it more convenient applying the normalized reduced probability density in proving the theorem for the Rényi entropy: the Rényi entropy $R^{\alpha }$ of the ground state density is a strictly monotonous (increasing or decreasing) function of the control parameter ξ in a neighborhood of the transition point $\xi_{c}$, and its derivative $dR^{\alpha }/d\xi$ diverges at $\xi_{c}$ in the thermodynamic limit. There is also a one-to-one map between the Rényi entropy of a given order and the ‘external potential’. That is, it has been established that the Rényi entropy, for a given order, may be employed as an alternative *control parameter*. It has likewise been shown that degenerate cases can be treated in an analogous manner by means of the *subspace density*.

In light of this structuring role, established a rigorous relationship between the *relative Rényi entropy* and the *fidelity susceptibility* has been established [28], demonstrating that the former is, to a very good approximation, proportional to the latter and that the relative Rényi entropy coincides with the Kullback–Leibler entropy multiplied by the Rényi parameter. The usefulness of these kinds of information has been exhibited by the Dicke model as it is detailed in the following section.

## 3. Relative Rényi Entropy and Fidelity Susceptibility

Let *f* and *g* be probability functions. To quantify the dissimilarity of *f* from a reference density *g* (assume *f* is absolutely continuous with respect to *g*), the relative Rényi entropy is

(34) $$R_{f,g}^{\alpha }=\frac{1}{\alpha -1}\;\ln\;\int \frac{f^{\alpha }\left(r\right)}{g^{\alpha -1}\left(r\right)}\;dr,$$

for α>0 whenever the integral exists. As α→1, one obtains the Kullback–Leibler (KL) divergence [24]

(35) $$I_{KL}\left(f,g\right)=\int f\left(r\right)\;\ln\;\frac{f(r)}{g(r)}\;dr.$$

While $I_{KL}$ is not symmetric and does not satisfy the triangle inequality, it is central in asymptotic hypothesis testing and information geometry. The Rényi family strengthens or relaxes sensitivity to the distribution tails depending on α, often sharpening detection of changes in systems approaching a phase transition.

### 3.1. Quantum Fidelity and a DFT-Based Fidelity Between Densities

Fidelity proved to be a significant quantity in QPT. We refer to the excellent review on the fidelity approach by Gu [29]. For pure quantum states |Φ〉 and |Ψ〉, fidelity is the overlap

(36) F(Φ,Ψ)=|〈Φ|Ψ〉|,

which quantifies state distinguishability (F=1 for identical states; F=0 for orthogonal ones). In density-functional contexts where the basic descriptor is a density rather than a wavefunction it is natural to consider a fidelity directly on densities:

(37) $$f_{e,\sigma }=\int \sqrt{e(\tau )\;\sigma (\tau )}\;d\tau ,$$

with $0\leq f_{e,\sigma }\leq 1$ and equality 1 iff e=σ almost everywhere. For many-variable wavefunctions Φ,

(38) $$e\left(q\right)={\left|\Phi \left(q\right)\right|}^{2},\;e\left(q\right)=\int {\left|\Phi \left(q,\tau \right)\right|}^{2}\;d\tau ,$$

where e(q) can represent a marginal (reduced) density obtained by integrating out degrees of freedom. In practice, comparing reduced densities is often sufficient to detect structural changes of the ground state.

### 3.2. Fidelity Susceptibility and Fisher Information

Consider $H\left(\lambda \right)=H_{0}+\lambda V$ with control parameter λ. Near λ, the ground-state fidelity expands as [15,29]

(39) $$F\left(\lambda ,\lambda +\delta \lambda \right)=1-\frac{1}{2}{\left(\delta \lambda \right)}^{2}\;\chi_{F}+\ldots ,$$

where $\chi_{F}$ is the fidelity susceptibility, a curvature of the fidelity landscape that typically peaks or diverges at criticality. Analogously, the density-based fidelity expands as

(40) $$f\left(\lambda ,\lambda +\delta \lambda \right)=1-\frac{1}{2}{\left(\delta \lambda \right)}^{2}\;\chi_{f}+\ldots ,$$

with

(41) $$\chi_{f}=\frac{1}{4}\int \frac{1}{e(\tau )}\;{\left(\frac{\partial e}{\partial \lambda }\right)}^{\;2}d\tau .$$

The quantity $\chi_{f}$ is proportional to the (classical) Fisher information [2],

(42) $$I=\int \frac{1}{e(x)}\;{\left(\frac{\partial e}{\partial \lambda }\right)}^{\;2}dx,$$

which measures the statistical sensitivity of the family e(·;λ) to parameter changes. Large *I* signals that even a small parameter shift produces a noticeable redistribution of probability mass-behavior expected near a quantum critical point.

Expanding the density in δλ,

(43) $$e\left(\lambda +\delta \lambda \right)=e\left(\lambda \right)+\delta \lambda \;\frac{\partial e}{\partial \lambda }+\frac{{\left(\delta \lambda \right)}^{2}}{2}\frac{\partial^{2}e}{\partial \lambda^{2}}+\ldots ,$$

and using normalization,

(44) $$\int e\left(\lambda \right)\;d\tau =1,\;\int \frac{\partial e}{\partial \lambda }\;d\tau =\frac{\partial }{\partial \lambda }\int e\left(\lambda \right)d\tau =0,$$

the linear term integrates to zero, and the quadratic term is controlled by *I* in (42). Under mild smoothness and integrability (dominated convergence), the leading-order relation is

(45) $$R_{e(\lambda ),e(\lambda +\delta \lambda )}^{\alpha }\approx \frac{1}{1-\alpha }\ln\;\left[1+\frac{1}{2}\alpha \left(1-\alpha \right)\;I\;{\left(\delta \lambda \right)}^{2}\right].$$

For sufficiently small ${\left(\delta \lambda \right)}^{2}$, using ln(1+x)≈x yields [28]

(46) $$R_{e(\lambda ),e(\lambda +\delta \lambda )}^{\alpha }\;\approx \;\frac{\alpha }{2}\;I\;{\left(\delta \lambda \right)}^{2}.$$

Thus, relative Rényi entropy provides a quadratic, information-geometric measure of local change along the λ manifold of densities.

### 3.3. KL Limit and Proportionality to Rényi

Taking α→1 recovers a KL-based relation,

(47) $$I^{KL}\;\left(e(\lambda ),e(\lambda +\delta \lambda )\right)\;\approx \;2\;\chi \;{\left(\delta \lambda \right)}^{2},$$

so that, at leading order [28],

(48) $$R_{e(\lambda ),e(\lambda +\delta \lambda )}^{\alpha }\;\approx \;\alpha \;I^{KL}\;\left(e(\lambda ),e(\lambda +\delta \lambda )\right).$$

This formalizes the statement that, in the local (small-shift) regime, Rényi divergences rescale the KL divergence by the order α.

### 3.4. Linear Relation to Density Fidelity and a Quality Factor

Because 1−f is a second-order dissimilarity measure, the relative Rényi entropy is linearly related to the density fidelity deficit [28]:

(49) $$\frac{1}{4\alpha }\;R_{e(\lambda ),e(\lambda +\delta \lambda )}^{\alpha }\;\approx \;1-f\left(\lambda ,\lambda +\delta \lambda \right)\;\approx \;\frac{1}{2}\;\chi \;{\left(\delta \lambda \right)}^{2}.$$

The approximation is typically excellent away from the immediate vicinity of the critical point, where *I* may become large and higher-order terms non-negligible. A convenient diagnostic is the dimensionless quality factor [28]

(50) $$C^{\left(\alpha \right)}\left(\lambda ,\delta \lambda \right)\;\equiv \;\frac{f(\lambda ,\lambda +\delta \lambda )}{\;1-R_{e(\lambda ),e(\lambda +\delta \lambda )}^{\alpha }/\left(4\alpha \right)\;},$$

which remains close to unity in the perturbative regime and deviates near criticality, signaling the breakdown of the quadratic approximation. Differentiating with respect to α gives another identity useful for sensitivity analyses [28]:

(51) $$\frac{\partial R_{e(\lambda ),e(\lambda +\delta \lambda )}^{\alpha }}{\partial \alpha }\;\approx \;\frac{1}{2}\;I\;{\left(\delta \lambda \right)}^{2}\;=\;I^{KL}\;\left(e(\lambda ),e(\lambda +\delta \lambda )\right).$$

To highlight the usefulness of the connection between fidelity and relative Rényi information, we focus on the Dicke model—a paradigmatic spin–boson system. It has long served as a benchmark for studies of quantum–optical behavior, quantum chaos, and entanglement (see [16,17,30,31,32,33,34,35]). It is also worth emphasizing that direct experimental realizations of the Dicke model have been demonstrated in recent years [36,37].

The *Dicke model* formalizes the interaction between a single electromagnetic mode of frequency ω and an ensemble of *N* two-level emitters separated by an energy $\omega_{0}$. The strength of the dipolar coupling is controlled by the parameter λ[16,17,30,31]. In terms of the field bosonic operators $a,a^{†}$ and the generators $J_{z},J_{\pm }$ of a pseudospin of length j=N/2, the Hamiltonian takes the form

(52) $$H\;=\;\omega_{0}J_{z}\;+\;\omega \;a^{†}a\;+\;\frac{\lambda }{\sqrt{2j}}\;\left(a^{†}+a\right)\;\left(J_{+}+J_{-}\right),$$

which is the canonical version of an optical field collectively coupled to an atomic “cloud”.

This system exhibits a *quantum phase transition* in the thermodynamic limit N→∞ (equivalently j→∞): upon crossing the critical value $\lambda_{c}=\sqrt{\omega \;\omega_{0}}/2$ the ground state changes from the *normal phase* ($\lambda <\lambda_{c}$) to the *superradiant phase* ($\lambda >\lambda_{c}$) [32,33,34]. Moreover, the model possesses a *parity symmetry* generated by $\hat{\Pi }=e^{i\pi \;(a^{†}a+J_{z}+j)}$, which commutes with the Hamiltonian, $[\hat{\Pi },H]=0$ [17,35].

For numerical analyses it is convenient to work in the product basis {|n;j,m〉}≡|n〉⊗|j,m〉, where |n〉 denotes photon-number states, and |j,m〉 are the Dicke states of the atomic sector. An arbitrary state vector can thus be written as

(53) $$|\psi \rangle \;=\;\sum_{n=0}^{n_{c}}\;\sum_{m=-j}^{j}c_{nm}^{\left(j\right)}\;|n;j,m\rangle ,$$

with coefficients $c_{nm}^{\left(j\right)}$ determined variationally or exactly after a bosonic *truncation* to $n\leq n_{c}$.

The *position-space density* is obtained from the wave function ψ(x,y) as

(54) $$\varrho \left(x,y\right)\;=\;|\psi \left(x,y\right){|}^{2},$$

and, in position representation, the wave function reads

(55) $$\psi \left(x,y\right)\;=\;e^{-\frac{1}{2}\left(\omega x^{2}+\omega_{0}y^{2}\right)}\sum_{n=0}^{n_{c}}\;\sum_{m=-j}^{j}\frac{H_{n}\left(\sqrt{\omega }\;x\right)\;H_{j+m}\left(\sqrt{\omega_{0}}\;y\right)}{2^{(n+m+j)/2}\;\sqrt{n!\;(j+m)!}}\;c_{nm}^{\left(j\right)},$$

where we have used the position eigenfunctions of the field oscillator,

(56) $$\langle x|n\rangle \;=\;\omega^{1/4}e^{-\frac{1}{2}\omega x^{2}}\;\frac{H_{n}\left(\sqrt{\omega }\;x\right)}{\sqrt{2^{n}n!\;\sqrt{\pi }}}\;,$$

as well as those of the atomic sector in the Holstein–Primakoff approximation (see, e.g., [17])

(57) $$\langle y|j,m\rangle \;=\;\omega_{0}^{1/4}e^{-\frac{1}{2}\omega_{0}y^{2}}\;\frac{H_{j+m}\left(\sqrt{\omega_{0}}\;y\right)}{\sqrt{2^{\left(j+m\right)}\left(j+m\right)!\;\sqrt{\pi }}}\;.$$

**Figure 1:**
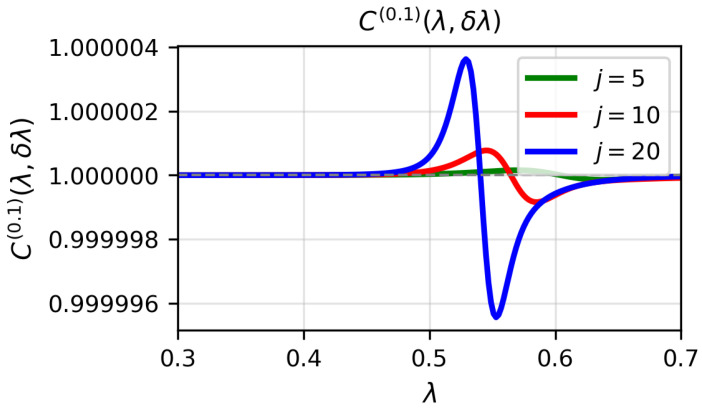
(Colour on-line) $C^{\left(0.1\right)}\left(\lambda ,\delta \lambda \right)$ for δλ=0.002 for j=5,10,20 and $\omega_{0}=\omega =1$ as a function of λ for the numerical ground-state wave function. Atomic units are used.

**Figure 2:**
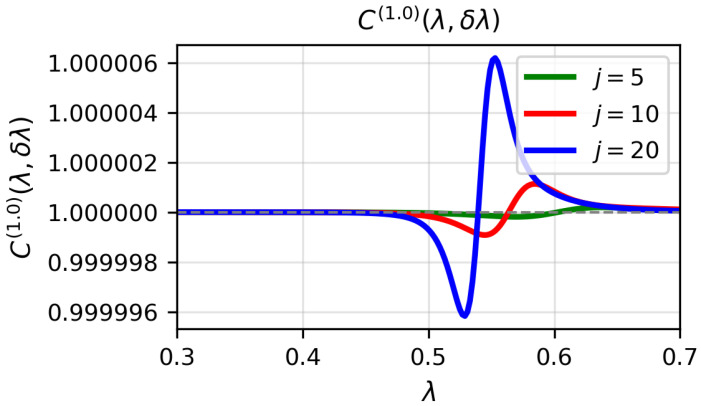
(Colour on-line) $C^{\left(1.0\right)}\left(\lambda ,\delta \lambda \right)$ for δλ=0.002 for j=5,10,20 and $\omega_{0}=\omega =1$ as a function of λ for the numerical ground-state wave function. Atomic units are used.

**Figure 3:**
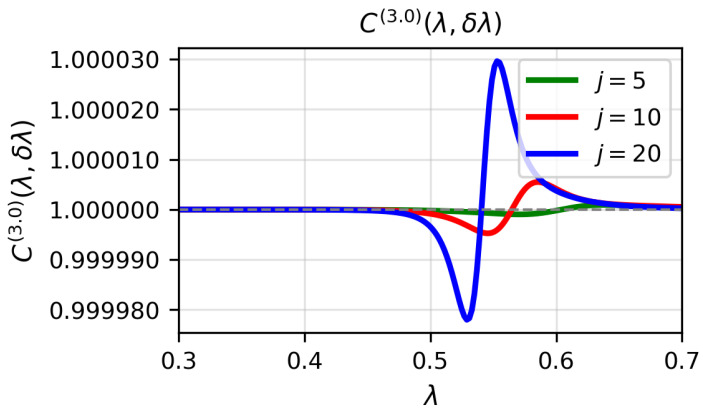
(Colour on-line) $C^{\left(3.0\right)}\left(\lambda ,\delta \lambda \right)$ for δλ=0.002 for j=5,10,20 and $\omega_{0}=\omega =1$ as a function of λ for the numerical ground-state wave function. Atomic units are used.

Figure 1, Figure 2 and Figure 3 display the quantity $C^{\left(\alpha \right)}\left(\lambda ,\delta \lambda \right)$ for α=0.1, α=1 and α=3, respectively, as a function of λ for j=5,10,20, with δλ=0.002. In the numerical calculations we set the bosonic truncation cutoff to $n_{\max}=14$, retaining Fock states up to n=14. The approximation $C^{\left(\alpha \right)}\;\approx 1$ is excellent far from the critical point, but it deteriorates as λ approaches $\lambda_{c}$. The difference $C^{\left(\alpha \right)}\left(\lambda ,\delta \lambda \right)-\left(1-F(\lambda ,\lambda +\delta \lambda )\right)$ becomes more pronounced with increasing *j*, reflecting the fact that the critical region sharpens with spin size.

We note that the Holstein–Primakoff representation is a large-*j* approximation and may become less accurate very close to criticality due to enhanced fluctuations; in our work it is used only to guide the interpretation, while the numerical results are obtained from exact diagonalization in the full (2j+1)-dimensional atomic sector.

## 4. Discussion and Summary

Information concepts have a growing importance in several fields of physics and chemistry. They proved to be especially significant in DFT due to the fact that the density can be considered a probability density if it is normalized to 1 (Usually, the integral of the density gives the number of electrons in DFT). As DFT has been extended to treat QPT, it is reasonable to apply information concepts in QPT, too. Here, the efficiency of these kinds of information is illustrated by the Dicke model. There are, however, several other examples, where information approaches appeared beneficial. The main computational advantage is that these Rényi-based quantities can be constructed directly from densities or other reduced distributions, without requiring explicit access to the many-body wave function. In large-scale simulations, computing such reduced objects is typically far less demanding (in both memory and runtime) than manipulating the full state or evaluating wave-function overlaps. Consequently, density-based information-theoretic diagnostics provide a scalable criterion to detect rapid changes of the ground state and to identify candidate critical regions with reduced computational overhead.

It is worth mentioning that DFT can enlighten phase transitions at finite temperatures and give new insight into its connection with entanglement [38]. Wei [38] showed that there is a one-to-one map between the set of the thermal equilibrium densites and set of the control parameters in thermal equilibrium with temperature *T*, where the entropy *S* is added to the set of densites and the temperature *T* is added to the set of control parameters. Moreover, any finite temperature entanglement measure can be expressed as a unique functional of the set of first derivatives of the free energy.

According to DFT the density contains sufficient information to compute the value of any observable. It has recently been shown [39] that the Fisher information density also incorporates this knowledge, so a Fisher information density functional theory has been constructed. It has also been shown that the Shannon entropy density can be used as a descriptor of a Coulomb system.

In this review we summarised how DFT provides a conceptual and efficient scheme of treating QPT. Within the framework of DFT, where the *density* serves as the fundamental variable, there exists a direct analogue for the analysis of QPT. It has likewise been shown that degenerate cases can be treated in an analogous manner by means of the *subspace density*. The fact that the control parameter can be considered a DFT-like external potential and the *density* can be obtained from the Hellmann–Feynman theorem has turned out to be fruitful in demonstrating that there is a one-to-one map between the non-degenerate wave function and the control parameter (in degenerate case between the subspace spanned by the degenerate wave function and the control parameter). Moreover, a new *density* with different control parameter can be obtained employing any strictly monotonous function. Hohenberg–Kohn-like theorems have been proven to be valid.

We studied among others Rényi entropy and found that there is a one-to-one map between the Rényi entropy and the *external potential*; therefore, the Rényi entropy may be employed as an alternative *control parameter*. A rigorous relationship between the *relative Rényi entropy* and the *fidelity susceptibility* has also been established. A dimensionless quality factor constructed from the relative Rényi entropy and the fidelity has proved to be a convenient indicator around the critical point.

Although in this work we use the Dicke model as a representative example, the DFT-based formulation and the information-theoretic diagnostics are readily applicable to other families of Hamiltonians of the form $\hat{H}\left(\xi \right)={\hat{H}}_{0}+\sum_{i}\xi_{i}{\hat{A}}_{i}$, where the expectation values $a_{i}=\langle {\hat{A}}_{i}\rangle$ act as variables conjugate to the control parameters. In strongly correlated lattice systems (e.g., Hubbard- and spin-type models), couplings such as the interaction strength, chemical potential, external fields, or anisotropies naturally correspond to operators ${\hat{A}}_{i}$ (double occupancy, magnetization, bond or current operators), and quantum phase transitions can be monitored through the behaviour of $a_{i}$, their susceptibilities, and information-theoretic measures constructed from accessible reduced distributions (real- or momentum-space densities, structure factors, or local reduced density matrices). Similarly, in quantum-simulation settings and topological phases one may define the same diagnostics using reduced objects sensitive to topology (e.g., edge-mode occupations or subsystem reduced density matrices), enabling the identification of rapid ground-state changes—including topological transitions.

In conclusion, the available body of results shows that information-theoretic concepts, when embedded within the DFT framework, provide a unified, highly sensitive, and conceptually transparent approach to the study of quantum phase transitions. Entropic and information measures such as Rényi and Wehrl entropies, Fisher information, participation ratios, complexity indicators, and fidelity-based quantities not only capture the abrupt restructuring and (de)localization of the wave function in paradigmatic many-body systems but also serve as sharp markers of topological transitions in gapped two-dimensional Dirac materials, faithfully tracking changes in the underlying topological invariants. At the same time, the DFT perspective clarifies why these tools are so powerful: the density (or subspace density in degenerate cases) and its associated information measures contain all the information required to reconstruct observables, to describe phase transitions at both zero and finite temperature, and to formulate Hohenberg–Kohn-type mappings in which the control parameter, viewed as an effective external potential, can be replaced by suitable informational quantities. In particular, the one-to-one correspondence between Rényi entropies and the external potential justifies their use as alternative control parameters, while the rigorous link between relative Rényi entropies and the fidelity susceptibility, together with the corresponding dimensionless quality factor, yields compact and robust indicators of criticality across a broad class of quantum and topological systems. 

## Data Availability

Data are available upon request.
